# Detecting EGFR Gene Mutations on a Nanobioarray Chip

**DOI:** 10.3390/biomedicines14010142

**Published:** 2026-01-10

**Authors:** Fang Xu, Montek Boparai, Christopher Oberc, Paul C. H. Li

**Affiliations:** Department of Chemistry, Simon Fraser University, Burnaby, BC V5A 1S6, Canada

**Keywords:** gene mutation, lung cancer, DNA hybridization, gold nanoparticle wash, fluorescence detection

## Abstract

In this study, three point mutations of EGFR relevant to lung cancer therapy are detected. Mutated EGFR is the target of a therapy for non-small cell lung cancer (NSCLC) using tyrosine kinase inhibitors (TKIs) as treatment drugs. **Background/Objectives:** Point mutations in exon 21 (L858R and L861Q) of the EGFR gene are TKI-sensitive; however, mutations in exon 20 (T790M) are TKI-resistant. Therefore, a fast detection method that classifies an NSCLC patient to be drug sensitive or drug resistant is highly clinically relevant. **Methods:** Probes were designed to detect three point mutations in genomic samples based on DNA hybridization on a solid surface. A method has been developed to detect single nucleotide polymorphism (SNP) for these mutation detections in the 16-channel nanobioarray chip. The wash by gold-nanoparticles (AuNP) was used to assist the differentiation detection. **Results:** The gold nanoparticle-assisted wash method has enhanced differentiation between WT and mutated sequences relevant to the EGFR sensitivity to tyrosine kinase inhibitors. **Conclusions:** The WT and mutated sequences (T790M, L858R and L861Q) in genomic samples were successfully differentiated from each other.

## 1. Introduction

EGFR (Epidermal Growth Factor Receptor) is a transmembrane protein; this is a receptor for the family of extracellular protein ligands called the epidermal growth factor [1]. Mutated EGFR is the target of a therapy using tyrosine kinase inhibitors (TKIs) for drug treatment of non-small cell lung cancer (NSCLC) [2]. TKIs, such as gefitinib and erlotibib, are recommended drugs for such a targeted therapy because they can remarkably prolong the median survival time of patients [3]. Mutations in exons 18, 19 and 21 of the EGFR gene are TKI-sensitive; however, mutations in exon 20 are drug-resistant [4]. Among these mutations detected by DNA sequencing, exon 20 has a T790M point mutation, and exon 21 has the L858R and L861Q point mutations [5]. Yamamoto suggests that a 42.7% chance of mutation would happen in exon 21, in particular in the form of L858R [6]. Therefore, we believe the detection of point mutations of T790M will cover most cases of the TKI-resistant phenotypes, and L858R and L861Q of the TKI-sensitive ones, and such a detection by DNA hybridization is the focus of the present paper.

The gold-standard detection method of point mutations is DNA sequencing [7,8,9], but this method is expensive and requires several days for sample preparation, sequencing reaction and data interpretation. Mutation detection by DNA hybridization is faster and less costly than by DNA sequencing for routine detection of point mutations [10,11,12]. In particular, our group has developed the use of gold-nanoparticles (AuNP) in a nanobioarray chip to facilitate such a detection by DNA hybridization [13,14]. In this paper, detections of the mutations relevant to the EGFR gene that occur in exon 20 (T790M) and exon 21 (L858R and L861Q) are conducted on genomic DNA samples in the 16-channel chip.

## 2. Materials and Methods

### 2.1. Microfluidic Device Fabrication

The procedures to fabricate a poly(dimethylsiloxane) (PDMS) chip have been introduced previously [15]. Briefly, the 16-channel chip design was printed on a transparency which later served as a photomask to make the mold for chip fabrication. To achieve this, the photomask was placed on a SU-8 (as the molding layer) coated silicon wafer for photolithography. After SU-8 development, a mold is formed, and the PDMS prepolymer was then poured onto the mold to produce the chip. The chip was 2 in × 2 in and was ~2 mm in thickness. The channels had the depth of 35 µm and width of 150 µm. The reservoirs were punched by a 1 mm diameter flat-end needle, and the PDMS slab was then sealed to an aldehyde-coated glass surface. To coat a glass slide (2 in × 3 in) with aldehyde functional groups, the slide was gently heated to boil in piranha solution for 15 min and rinsed. Then, it was immersed in 2% (3-aminopropyl)triethoxysilane (APTES) in ethanol (95% by volume) for 20 min, baked at 120 °C for 1 h, and finally treated with 5% (*v*/*v*) glutaraldehyde in phosphate-buffered saline (pH = 7.4) for 1 h.

### 2.2. DNA Sequences, Probes, Primers and 60-Mer Oligonucleotide Design

The DNA sequences relevant to exons 20 and 21 had been confirmed by three independent sources published in the National Center for Biotechnology Information (NM_001346941.1, NM005228.4, and NG_007726.3). From the sequence information, the probes, primers, and 60-mer oligonucleotides had been designed, as tabulated in Table 1, and they were synthesized and modified by International DNA Technologies (Coralville, IA, USA).

### 2.3. Genomic DNA and Polymerase Chain Reaction (PCR)

The genomic DNAs were purchased from Horizon Discovery (HD709, Cambridge, UK) (50 ng/µL) for the wild-type EGFR gene, and HD802 (50 ng/µL) for the mutated one. The mutated genomic DNA sample is a mixture of different mutations on exons 20 and 21.

The PCR mixtures contained 1x PCR buffer, 0.3 mM of each primer, 0.2 mM of each dNTP, 2 mM Mg^2+^, 0.1 U/µL Taq polymerase, and 0.5 ng/µL genomic DNA template. The PCR started with an initial denaturation step (94, 3 min), followed by 33 cycles of amplification with denaturation (95, 30 s) annealing (52, 30 s), and elongation (74, 30 s), and the PCR ended with a final elongation at 72 for 3 min.

The PCR products were then purified by a PCR purification kit (QIAquick, Qiagen, Toronto, ON, Canada), and their concentrations were determined by an ultraviolet (UV) spectrophotometer (NanoDrop 2000, Thermo Scientific, Mississauga, ON, Canada). The final PCR product was 185 bp long in exon 20, and 157 bp long in exon 21.

### 2.4. Probe Immobilization, Target Hybridization and Fluorescent Detection

The amine-labeled probes were diluted in 1.0 M NaCl and 0.15 M NaHCO_3_ (immobilization buffer). And the final 60-mer oligonucleotides and PCR product solutions were prepared in 0.015 M sodium citrate (C_6_H_5_Na_3_O_7_·2H_2_O), 0.15 M NaCl, and 0.1% sodium dodecyl sulfate (hybridization buffer).

**Figure 1:**
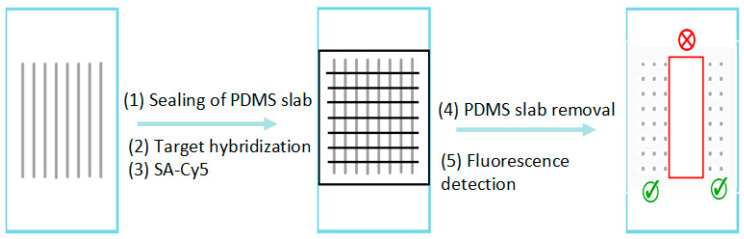
Schematic diagram for hybridization of labeled targets (horizontally aligned) on probes previously immobilized (vertically oriented) on an aldehyde-functionalized bioarray chip. The regions of proper hybridization are indicated with the green “check”; whereas the mismatch or no binding are depicted with the red “cross”.

The hybridization method conducted in a PDMS chip has been described in a previous published paper [16,17], which was based on the reaction between a probe DNA molecule and a labeled target DNA molecule. First, aminated probe solutions were immobilized onto an aldehyde-functionalized glass slide surface through a 16-channel PDMS slab that was sealed to the glass surface. After this first slab was removed, a second one was sealed to the same glass surface with the channels in the direction perpendicular to those of the first one (Figure 1). And then biotin-labeled target solutions (volume of 1 μL) were introduced through the channels. The targets hybridized at room temperature to the immobilized probe for 1 h. Post-hybridization, either a buffer wash or AuNP wash (5 nm) was conducted. It was previously reported that AuNP wash would improve differentiation [13,14,18]. For buffer wash, PBS was used. For AuNP wash, a solution containing 5 nM AuNP (5 nm) with 8 nM stabilizing oligonucleotide was used.

For fluorescence detection, the biotin on the target sequences were conjugated to streptavidin-cyanine 5 (SA-Cy5, 50 µg/mL), and the bounded sequences were detected by a phosphoimager (Typhoon 9410, GE Healthcare, Chicago, IL, USA), with the images further processed by ImageQuant 5.2.

## 3. Results and Discussion

### 3.1. Differentiation of ssDNA Samples

**Figure 2:**
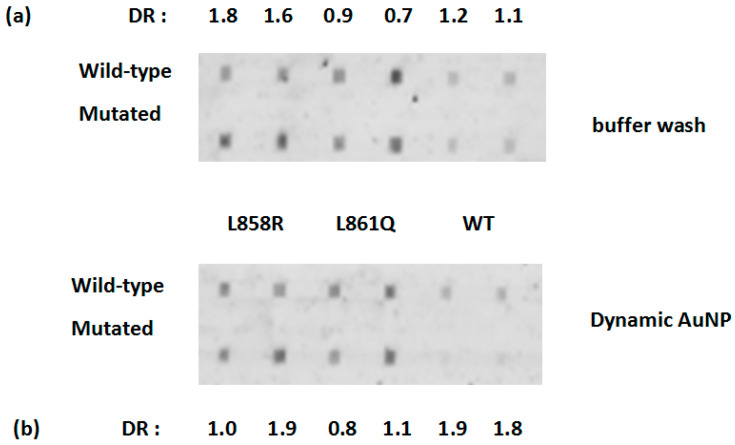
Differentiation of exon 21 mutations at room temperature at two probe concentrations (left 50 μM, right 75 μM, written as (75), (50), respectively), (**a**) buffer wash, (**b**) dynamic AuNP wash (3 s) with solution: 5 nm, 5 nM, 8 nM stabilizing DNA. The differentiation ratio (DR) was calculated by the fluorescent intensity of the perfect match (PM) duplex over mismatch (MM) duplex under the binding of each probe, with a DR value over 1.5 considered to be satisfactory. DR: differentiation ratio. The errors in DR are ±0.2.

The differentiation experiment of point mutations was first conducted at room temperature. The fluorescent image is depicted for the wild-type and mutated targets using probes immobilized with two different concentrations of probe solutions, see Figure 2. To evaluate binding specificity, we define the differentiation ratio (DR) which was calculated by the fluorescent intensity of the perfect match (PM) duplex over mismatch (MM) duplex under the binding of each probe, with a DR value over 1.5 considered to be satisfactory. With a post-hybridization buffer wash step, the differentiation of exon 21 mutations using L858R was satisfactory (DR = 1.8 for 50 μM probe and 1.6 for 75 μM probe) while the other two were unsatisfactory (L861Q: 0.9 and 0.7, WT: 1.2 and 1.1), see Figure 2a. The binding intensity towards the WT probe is quite weak, and this can be attributed to the secondary structures found in the 60-mer ssDNA, and in the probe. With the use of the dynamic AuNP wash, the DR did improve in WT (DR = 1.9 for 50 μM probe and 1.8 for 75 μM probe), but did not improve significantly for L858R (1.0 and 1.9) and L861Q (0.8 and 1.1), see Figure 2b.

**Figure 3:**
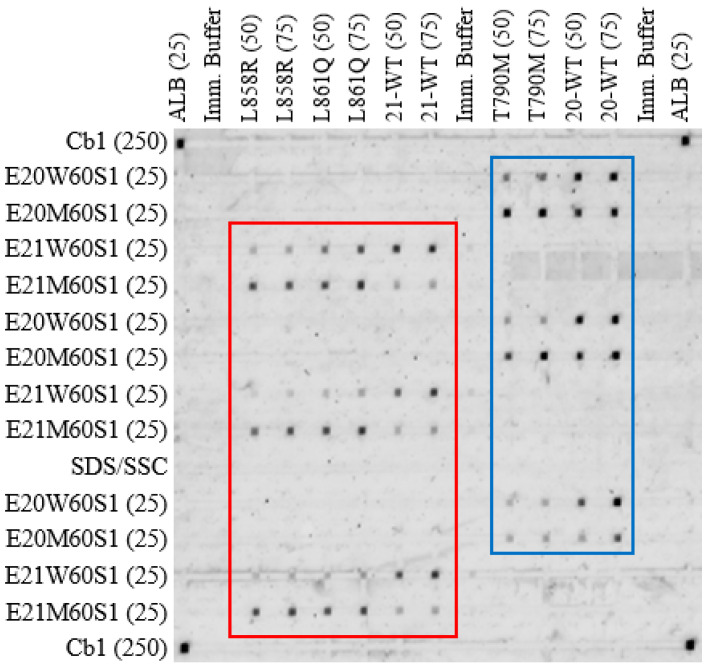
Differentiation of exon 20 and 21 mutations at 47 °C conducted on a 16 × 16 nanobioarray chip; AuNP wash solution: 5 nm, 5 nM AuNP with 8 nM stabilizing DNA. The boxed region (red box) for exon 21 was expanded and shown in Figure 4, whereas that for exon 20 (blue box) was shown in Figure 5.

**Figure 4:**
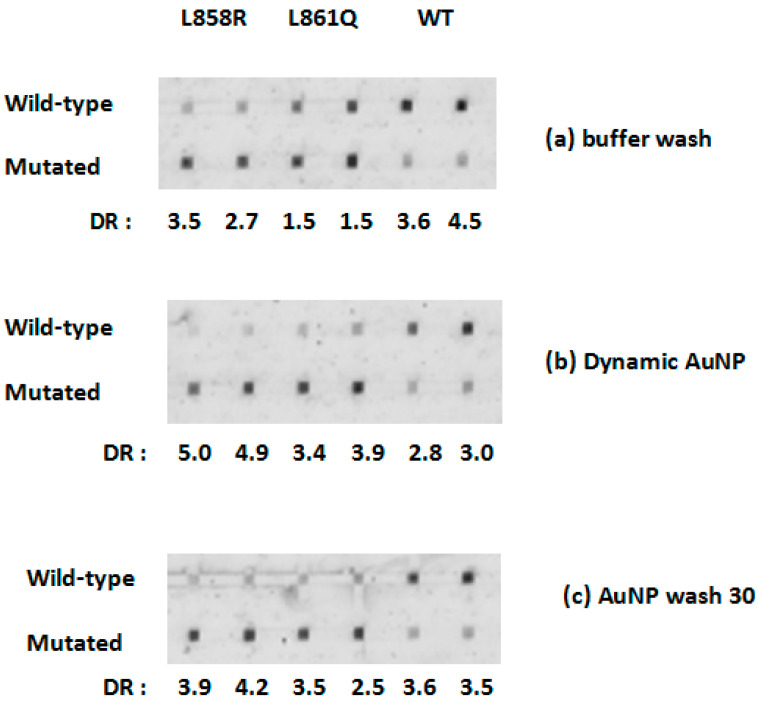
Differentiation of exon 21 mutations at 47 °C at two probe concentrations (left 50 μM, right 75 μM), (**a**) buffer wash, (**b**) AuNP wash (3 s), and (**c**) AuNP wash (30 min). DR: differentiation ratio. The errors in DR are ±0.2.

**Figure 5:**
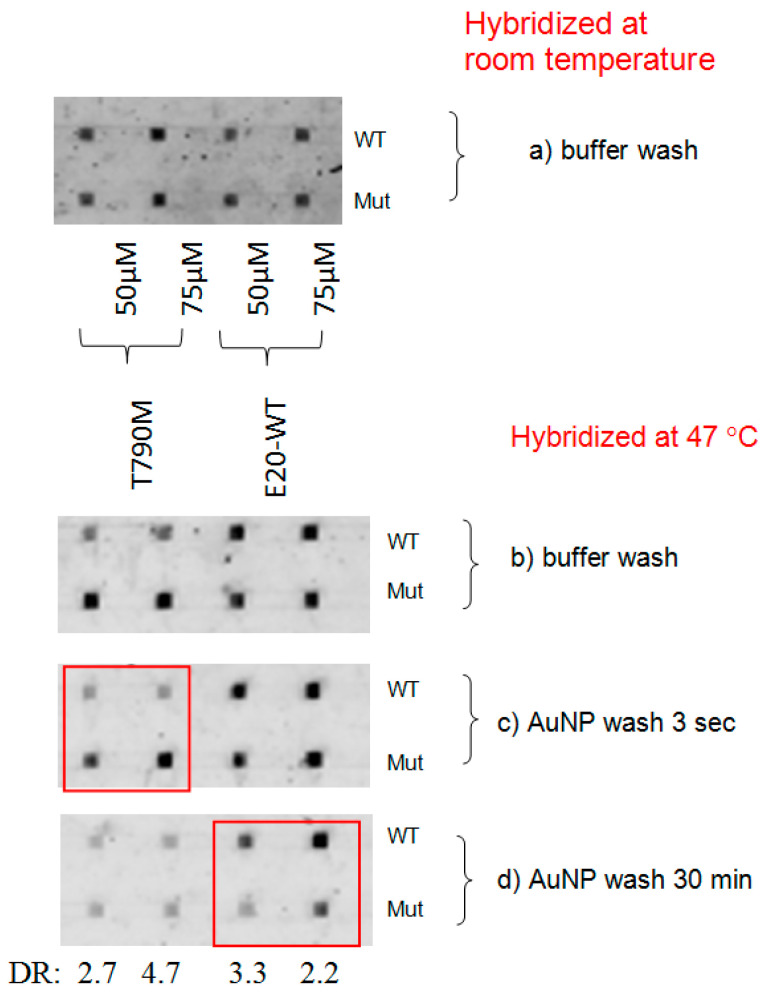
Differentiation of exon 20 mutations at two probe concentrations (left 50 μM, right 75 μM), (**a**) at room temperature with buffer wash, and at 47 °C followed by (**b**) buffer wash, (**c**) AuNP wash (3 s), and (**d**) AuNP wash (30 min). DR: differentiation ratio are given for the red-boxed regions. The errors in DR are ± 0.2.

To remove the effect of secondary structures, the experiment was repeated at 47 °C, see Figure 3. This temperature has been optimized in previous reports [14,16]. The results for the fluorescent image are expanded for exon 21 (Figure 4) and exon 20 (Figure 5). The elevated temperature resulted in significantly more intense signals for exon 21 WT, see Figure 4. Moreover, good DRs were seen under L858R (DR = 3.5 for 50 μM probe and 2.7 for 75 μM probe) and WT (DR: 3.6 and 4.5), but a marginal differentiation under L861Q (DR: 1.5 and 1.5), see Figure 4a. After the dynamic AuNP wash step was used, the mismatch intensities were decreased especially for L861Q, which resulted in higher DR, which are 3.5 and 2.5 for 50 μM and 75 μM probe, respectively, see Figure 4b. This indicates the use of both elevation in hybridization temperature and a subsequent dynamic AuNP wash has achieved satisfactory differentiation of the two point mutations in exon 21. The use of longer time for AuNP wash did not improve the DR values in a significant way, see Figure 4c.

For exon 20 mutation detection, the hybridization experiment conducted at 47 °C and AuNP wash resulted in good differentiation and DR values, as shown in Figure 5. For instance, DRs for T790M were good with the AuNP wash for 3 s, i.e., 2.7 and 4.7, see Figure 5c, as compared to Figure 5b with buffer wash and Figure 5a conducted at room temperature. Here, differentiation for WT was good with a longer-time AuNP wash, with DR values of 3.3 and 2.2, see Figure 5d.

**Figure 6:**
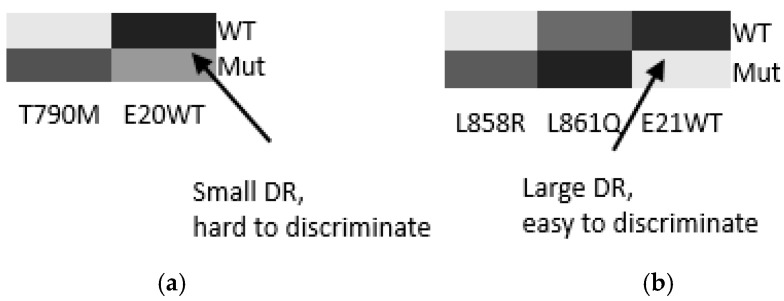
The heat map of different probe-target duplexes for (**a**) exon 20 and (**b**) exon 21. Differentiation ratio (DR) is calculated by the signal obtained in perfect matched against mismatched duplexes and depicted in grayscale in four levels such as black, dark gray, gray and white. For (**a**), the differentiation for E20WT is small; whereas for (**b**), the differentiation for E21WT is large.

The differentiation results can be partially predicted based on thermodynamic calculations at 47 °C, as shown by the heat map in Figure 6. Here, the differentiation is depicted in the grayscale to mimic the fluorescence images. The prediction results indicate the challenges of differentiation in WT (exon 20) and L861Q (exon 21). These challenges have been resolved by the use of AuNP wash, which invokes kinetic consideration, in addition to the equilibrium one.

### 3.2. Differentiation of PCR Strands

**Figure 7:**
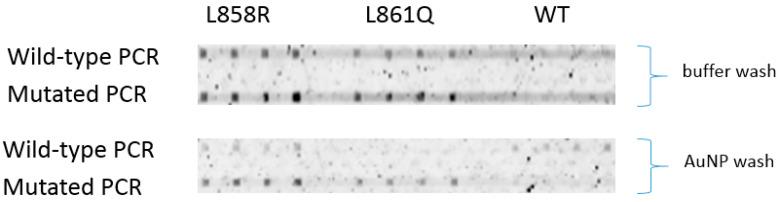
Hybridization of PCR products (157 bp); all PCR solutions (volume of 1 μL) were 25 nM; denatured before reaction; hybridization time: 10 min.

Detection of PCR strands was achieved with wild-type and mutated genomic DNA. At room temperature, hybridization signals of L858R and L861Q probes to the mutated PCR strand are higher than to the wild-type strand, especially when AuNP wash was used (Figure 7). Binding of wild-type PCR strand to WT probe is non-obvious with no AuNP wash because of a high background. But with AuNP wash, the fluorescence signal is seen from the wild-type PCR strand which indicates successful binding of wild-type PCR strand by the WT probe. Improvement is expected when hybridization is conducted at 47 °C.

## 4. Conclusions

We have detected DNA target sequences for EGFR gene mutation in exons 20 and 21, using the developed DNA hybridization method in a nanobioarray chip. PCR products were produced from genomic wild type (WT) and mutated DNA and applied to a 16-channel nanobioarray chip. The gold nanoparticle-assisted wash method was used to enhance differentiation between WT and mutated sequences relevant to the EGFR sensitivity to tyrosine kinase inhibitors. The WT and mutated sequences (T790M, L858R and L861Q) were successfully differentiated from each other.

This hybridization method is faster (i.e., in one day) and less expensive (i.e., less costly reagents and fast data processing) than the conventional method of DNA sequencing.

## Figures and Tables

**Table 1:** Probes, primers and 60-mer oligonucleotides for exon 20 and exon 21 detection. The bases which are affected by mutations had been underlined for both the WT and Mut sequences.

Acronym	Description	Length	Sequences (5′–3′) with Labels
E20_WT	Exon 20 WT probe	22	NH_2_-(CH_2_)_12_-CAT GAG CTG CGT GAT GAG CTG C
T790M	Exon 20 Mut probe	22	NH_2_-(CH_2_)_12_-CAT GAG CTG CAT GAT GAG CTG C
E21_WT	Exon 21 WT probe	21	NH_2_-(CH_2_)_12_-AGC AGT TTG GCC AGC CCA AAA
L858R	Exon 21 Mut site (1)	20	NH_2_-(CH_2_)_12_-TTG GCC CGC CCA AAA TCT GT
L861Q	Exon 21 Mut site (2)	21	NH_2_-(CH_2_)_12_-TCT TCC GCA CCC AGC TGT TTG
E20_F_Bio	Exon 20 Forward primer, biotin labeled	19	Bio-AAG CCT ACG TGA TGG CCA G
E20_R	Exon 20 Reverse primer	22	CTT TGC GAT CTG CAC ACA CCA G
E21_F_Bio	Exon 21 Forward primer, biotin labeled	22	Bio-GGG CAT GAA CTA CTT GGA GGA C
E21_R	Exon 21 Reverse primer	22	TTT GCC TCC TTC TGC ATG GTA T
E20W60	Exon 20, 60-mer oligonucleotides represent WT sequences, biotin labeled	60	Bio-CCT CAC CTC CAC CGT GCA GCT CAT CAC GCA GCT CAT GCC CTT CGG CTG CCT CCT GGA CTA
E20M60	Exon 20, 60-mer oligonucleotides represent Mut sequences, biotin labeled	60	Bio-CCT CAC CTC CAC CGT GCA GCT CAT CAT GCA GCT CAT GCC CTT CGG CTG CCT CCT GGA CTA
E21W60	Exon 21, 60-mer oligonucleotides represent WT sequences, biotin labeled	60	Bio-CAA GAT CAC AGA TTT TGG GCT GGC CAA ACT GCT GGG TGC GGA AGA GAA AGA ATA CCA TGC
E21M60	Exon 21, 60-mer oligonucleotides represent Mut sequences, biotin labeled	60	Bio-CAA GAT CAC AGA TTT TGG GCG GGC CAA ACA GCT GGG TGC GGA AGA GAA AGA ATA CCA TGC

## Data Availability

The original contributions presented in this study are included in the article. Further enquiries can be directed to the corresponding author.
